# LncRNA TCONS_00145741 Knockdown Prevents Thrombin-Induced M1 Differentiation of Microglia in Intracerebral Hemorrhage by Enhancing the Interaction Between DUSP6 and JNK

**DOI:** 10.3389/fcell.2021.684842

**Published:** 2022-01-19

**Authors:** Lanxiang Wu, Qingqing Zhan, Pan Liu, Heqing Zheng, Mingxu Liu, Jun Min, Liang Xie, Wei Wu

**Affiliations:** Department of Neurology, the Second Affiliated Hospital of Nanchang University, Institute of Neuroscience, Nanchang University, Nanchang, China

**Keywords:** TCONS_00145741, intracerebral hemorrhage, microglia, thrombin, JNK MAPK signaling pathway

## Abstract

**Background:** The differentiation of microglia from M1 to M2 exerts a pivotal role in the aggression of intracerebral hemorrhage (ICH), and long non-coding RNAs (lncRNAs) are associated with the differentiation of microglia. However, the underlying mechanism had not been fully clarified.

**Methods:** The expression profile of lncRNAs in thrombin-induced primary microglia was analyzed by RNA sequencing. Under thrombin treatment, the effect of lncRNA TCONS_00145741 on the differentiation of microglia was determined by immunofluorescence staining, quantitative real-time PCR, and Western blot. The potential mechanism and related signaling pathways of TCONS_00145741 in the M1 and M2 differentiation of microglia in ICH were assessed by Gene Ontology analysis, flow cytometry, RNA pull-down, RNA Immunoprecipitation, and RNA fluorescence *in situ* hybridization followed by immunofluorescence analysis.

**Results:** LncRNA TCONS_00145741 expression was elevated in the thrombin-induced primary microglia, and the interference with TCONS_00145741 restrained the M1 differentiation of microglia and facilitated the M2 differentiation under thrombin treatment. The interference with TCONS_00145741 restrained the activation of the JNK pathway in microglia under thrombin treatment and repressed the JNK phosphorylation levels by enhancing the interaction between DUSP6 and JNK. *In vivo* experiments further illustrated that the interference with TCONS_00145741 alleviated ICH.

**Conclusion:** LncRNA TCONS_00145741 knockdown prevented thrombin-induced M1 differentiation of microglia in ICH by enhancing the interaction between DUSP6 and JNK. This study might provide a promising target for the clinical treatment of ICH.

## Introduction

Intracerebral hemorrhage (ICH) accounts for about 10–20 percent of all strokes and has a high rate of death and disability (van Asch et al., 2010; Keep et al., 2012; An et al., 2017). After ICH occurs, the rapid accumulation of blood in the surrounding brain causes high pressure in the local brain tissue (Qureshi et al., 2009; Wang et al., 2015). Brain edema and neuroinflammation after ICH induce a series of secondary injuries, resulting in severe neurological impairment (Qureshi et al., 2001). At present, there is still a lack of effective treatment strategies for ICH and secondary brain injury caused by ICH. Thus, it is urgent to develop novel strategies to prevent ICH.

Microglia are widely recognized as the earliest inflammatory cells that respond to ICH (Wang, 2010; Yang et al., 2015a). Cumulative research clarifies that microglia differentiation exerts pivotal functions in the pathogenesis and progression of ICH (Qiao et al., 2018; Giordano et al., 2020). Upon activation, microglia polarize into different phenotypes that contribute differently to neuroinflammation in brain disease models (Walker and Lue, 2015). As has been reported, the M1 differentiation of microglia is generally considered to be a “classically activated” phenotype that accelerates the release of proinflammatory cytokines and increases reactive oxygen species (Lan et al., 2017a); M2 differentiation of microglia contributes to phagocytic debris and hematoma clearance (Lan et al., 2017b), and secretes anti-inflammatory cytokines and growth factors that have neuroprotective functions and facilitate neurological recovery (Zhou et al., 2020). Thus, repressing the M1 differentiation of microglia and facilitating the M2 differentiation contribute to the recovery of brain injury after ICH.

Recently, non-coding RNAs (ncRNAs) have been confirmed as clinical diagnostic or prognostic markers of ICH. Due to the key regulatory functions of long non-coding RNAs (lncRNAs) in various neurological diseases such as dementia, epilepsy, and cerebral ischemia, lncRNAs have attracted widespread attention (Lee et al., 2015; Kundap and Paudel, 2020; Stanzione et al., 2020). Previous studies clarify that lncRNAs exert momentous functions in ICH. For instance, Chen *et al.* corroborated that lncRNA Mtss1 facilitates inflammatory responses and secondary brain injury after ICH in mice (Chen et al., 2020); Wen *et al.* demonstrated that lncRNA Ptprj-as1 facilitates the secretion of inflammatory cytokines by elevating the proportion of M1 microglia and participates in the inflammatory injury caused by ICH (Wen et al., 2018). However, there is no related research on the changes of the lncRNA expression profile of microglia in thrombin-induced microglia. Thus, investigating the expressions of lncRNAs in ICH might provide novel targets for the mechanism of microglia in ICH.

Increasing evidence illustrates that thrombin facilitates secondary injury after ICH (Yang et al., 2015b; Krenzlin et al., 2020), and the influence of thrombin on the polarization of microglia has been confirmed (Wu et al., 2017). Therefore, we used an *in vitro* thrombin toxicity model for microglia to investigate the expression profile of lncRNAs based on thrombin-driven microglia activation and corroborated that TCONS_00145741, which ensemble is Ensmusg00000106219, locates on chromosome 5 and full-length is 2630 bp, was abnormally highly expressed and was interrelated to the regulation of M1 and M2 differentiation of microglia. Based on these findings, we further investigated the mechanism and related signaling pathways of TCONS_00145741 in M1 and M2 differentiation of microglia in ICH.

## Materials and Methods

### Construction of a Mouse Model of an Acute ICH

Twenty-four male C57BL/6 mice (8–10 weeks old) were provided from Cyagen (Suzhou, China). All mice were kept in an identical condition with a room temperature of 25°C and a 12h/12h light-dark cycle. Mice were free to obtain food and water.

Mice were randomly divided into four groups. Six mice were assigned to each group. For the mice in the ICH group, on the right side of the mouse’s brain +2 mm lateral to bregma and lower to the surface of the skull, we used a micro-injection needle to take 15 μl autologous blood and injected it into the brain, and the depth of the needle was 3.5 mm. Then, we injected 7.5 μl of blood at a rate of 1 μL/min. After nearly 5 min, we minimized the intracranial pressure of the mouse and injected the remaining 7.5 μl of blood at the same rate. Next, we covered the drilled hole with bone wax. For the mice in the sham group, other operations were the same as those in the ICH group except that no autologous blood was injected. Besides, the mice were euthanized at 6, 24, and 72 h after the establishment of the ICH model, and the brain tissues around the cerebral hemorrhage were isolated for subsequent assays. All animal experiments were approved by the Animal Care and Use Committee of the Second Affiliated Hospital of Nanchang University, Institute of Neuroscience, Nanchang University.

### Immunohistochemistry Assay

The brain tissues of mice were fixed with 10% formalin. Then, the fixed brain tissues were made into 2 mm slices. Next, the slices were permeated with 0.3% Triton X-100 for 30 min and then blocked with 5% bovine serum albumin (BSA) for 20–25 min, followed by the incubation with anti-thrombin (sc-271449, Santa Cruz Biotechnology) overnight at 4°C. The slices were further incubated with secondary antibodies for about 30 min at room temperature (RT). The images were acquired by a fluorescence microscope (Olympus OX51, Tokyo, Japan).

### Immunofluorescence

The immunofluorescence assay was conducted following the previously reported methods with minor changes (Wu et al., 2019). The paraffin-embedded slices were obtained in the same way as above. Similarly, the slices were permeated with 0.3% Triton X-100 for 30 min and blocked them with 5% BSA for 25 min, followed by the incubation with anti-Iba-1 (ab153696, 1:500, Abcam; sc-32725, 1:100, Santa Cruz Biotechnology), anti-CD86 (sc-28347, 1:100, Santa Cruz Biotechnology), anti-iNOS (ab178846, 1:500, Abcam), anti-CD206 (60143-1-1g, 1:100, Proteintech), anti-CD163 (ab182422, 100, Abcam) and anti- p-JNK (sc-6254, 1:200, Santa Cruz Biotechnology) overnight at 4°C. The slices were then incubated with the secondary antibodies for nearly 30 min at RT. Cell nuclei were stained using 4′,6-diamidino-2-phenylindole (DAPI, Thermo Fisher Scientific, MA, United States). The “sham”, “control” and “vehicle” were applied as the controls. The images were acquired by a fluorescence microscope (Olympus OX51).

### Isolation and Culture of Mouse Primary Microglia

Based on the previously described methods with minor modifications (Lan et al., 2017a), we performed the isolation and culture of primary microglia from male C57BL/6 mice (8–10 weeks old). Briefly, we isolated the glial cells from the brains of mice and then placed the cells (1  ×  10^6^ cells/ml) in the poly-D-lysine Dulbecco’s modified eagle medium (DMEM) medium containing 20% fetal bovine serum (FBS) and 1% antibiotic antimycotic solution, followed by the culture of them at 37°C, 5% CO_2_. We replaced the fresh culture medium every 2–3 days. When the mixed glial cells converge (nearly 14 days), we isolated microglia from the mixed glial population. In this study, we used microglia cultures with a purity of over 98%.

### Cell Culture, Thrombin Treatment and Differentiation

Human Microglial Clone 3 (HMC3) cells were from Procell (Wuhan, China). The cells were put in a minimum essential medium (MEM) (Thermo Fisher Scientific) containing 10% FBS and 1% penicillin and streptomycin (Thermo Fisher Scientific) and then maintained at 37°C, 5% CO_2_.

To investigate the expression profile of lncRNAs in primary mouse microglia treated with thrombin, the primary mouse microglia were treated with 20 U/ml thrombin for 24 h.

To explore the effect of TCONS_00145741 on the differentiation of microglia cell lines BV2 or primary microglia under thrombin treatment, BV2 cells transfected with si-TCONS_00145741 were treated with 20 U/ml thrombin for 24 h; BV2 cells transfected with pcDNA-TCONS_00145741 were treated with 10 ng/ml interleukin (IL)-4 and 10 ng/ml IL-13 for 24 h; primary microglia transfected with si-TCONS_00145741 were treated with 20 U/ml thrombin for 24 h.

To investigate the effect of TCONS_00145741 on the activation of the JNK pathway in microglial cell lines BV2 or primary microglia under thrombin treatment, BV2 cells and primary microglia transfected with si-TCONS_00145741 were treated with 20 μM thrombin, and then the cells were treated with 3 μM or 20 μM JNK pathway inhibitor SP600125.

### LncRNA Sequencing by Illumina HiSeq

TRIzol reagent (Invitrogen)/RNeasy MiniKit^®^ (Qiagen) was carried out to extract total RNA from primary mouse microglia treated with 20 U/ml thrombin. 1% agarose gels were conducted to quantify the contamination and degradation of total RNA, and RNA was quantified using Agilent 2100 bioanalyzer (Agilentgil Technologies, Palo Alto, CA, United States) and NanoDrop (Thermo Fisher). 1 μg total RNA with RIN value above 7 was chosen for the library preparation. Next-generation sequencing library preparations were constructed based on the protocol of the manufacturer (NEBNext^®^ Ultra™ Directional RNA Library Prep Kit for Illumina^®^), and the LncRNA sequencing was conducted by Illumina HiSeq.

### Quantitative Real-Time PCR

Referring to the previously described methods (Hui et al., 2019), we conducted the qRT-PCR assay. In brief, primary microglia and mouse microglia BV2 cells and brain tissues of mice after ICH 24 h were collected. We applied TRIzol to isolate total RNA. Then, the total RNA was reverse transcribed into cDNA using a PrimescriptTM RT kit (Takara, Beijing, China). Next, we conducted the real-time PCR using SYBR Premix Ex Taq™ (Takara) on an ABI 7500 Real-Time PCR System (Perkin-Elmer Applied Biosystems, United States). Glyceraldehyde-3-phosphate dehydrogenase (GAPDH) was applied as an internal reference and the relative expression was quantified using the 2^−ΔΔCT^ method. The primer sequences were exhibited in Table 1.

### Cell Transfection

After culturing BV2 cells to nearly 75% fusion, the synthetic si-TCONS_00145741 (abbreviated as si-741), si-TCONS_0026294 (abbreviated as si-294), si-TCONS_00094006 (abbreviated as si-006), 741 OVE (the overexpression of 741), and DUSP6 OVE (the overexpression of DUSP6) was transfected into BV2 cells using Lipofectamine 2000 Transfection Reagent (Invitrogen) based on the manufactures’ instructions, followed by the treatment of thrombin.

After the primary microglia were cultured to nearly 75% fusion, the synthetic si-741 was transfected into primary microglia using Lipofectamine 2000 referring to the manufactures’ instructions, followed by the treatment of thrombin. The sequences were displayed: si-NC (negative control): TTC​TCC​GAA​CGT​GTC​ACG​TCT; si-741: TTC​TAT​ATA​AGA​GAG​TCT​TAA​GG; si-294: CTC​GAA​AAA​CTA​CTA​ATA​GTA​AT; si-006: GTG​ATT​GCT​AGG​AAG​TAG​AAA​AG.

### Western Blot

Western blot assay was carried out as the previously described methods (Roman et al., 2020). Primary microglia and mouse microglia BV2 cells with different treatments were collected, and the total proteins were isolated using RIPA buffer (Gibco, United States). Then, the different protein samples were loaded into the lane of sodium dodecyl sulfate-polyacrylamide gel electrophoresis (SDS-PAGE) gels, followed by the transfer into polyvinylidene fluoride (PVDF) membranes (Sigma-Aldrich, United States). Next, the membranes were placed in 5% skim milk and blocked at RT for 1 h and then incubated with the specific primary antibodies at 4°C overnight. The antibodies were: anti-CD86 (ab243887, 1:1000, Abcam), anti-CD206 (ab252921, 1:1000, Abcam), anti-p-JNK (sc-6254, 1:500, Santa Cruz Biotechnology), anti-JNK(sc-7345, 1:500, Santa Cruz Biotechnology), anti-p-p38 (sc-166182, 1:500, Santa Cruz Biotechnology), anti-p38 (ab170099, 1:2000, Abcam), anti-pERK (sc-7383, 1:500, Santa Cruz Biotechnology), anti-ERK (ab32537, 1:1000, Abcam) and β-actin (ab6276, 1:5000, Abcam). The samples were further incubated with the secondary antibody (ab205718, 1:2000, Abcam) at 37°C for nearly 1 h. Ultimately, the enhanced chemiluminescence reagents (Millipore, Bedford, MA) and ImageJ were applied to analyze protein bands.

### Flow Cytometry

Flow cytometry was carried out to explore the effect of JNK pathway inhibitor SP600125 on the CD86 expression in microglia. Specifically, BV2 cells and primary microglia (1 × 10^5^ cells) were seeded in six-well plates. After treating BV2 cells and primary microglia with thrombin, the cells were incubated with different concentrations of JNK pathway inhibitor SP600125. Next, the above-mentioned cultured cells were collected, followed by the incubation with anti-CD206 (141707, BioLegend) and anti-CD86 (105011, BioLegend) for 30 min at RT. Ultimately, the cells were fixed and the data were collected using Millipore Guava EasyCyte 8 HT flow cytometer.

### Separation of Cytoplasm and Nucleus

BV2 cells and primary microglia (1 × 10^7^) were collected and were washed with pre-cooled PBS 2-3 times. Subsequently, the cells were mixed with cytoplasmic extraction buffer on ice for nearly 10 min and the lysate was transferred to a novel tube and was shaken vigorously for nearly 30 s, and then centrifuged at 4°C, 12,000 × rpm for 5–10 min. After that, the supernatant was immediately transferred to a novel pre-cooled test tube to obtain the cytoplasmic extract. Then, we resuspended the nuclear pellet in ice-cold nuclear lysis buffer and continued the incubation on ice for 60 s. Next, we spun the samples at 4°C, 14,000 rpm for 5–10 min. The supernatant of the spin was the nuclear extract and immediately transferred to a novel pre-cooled test tube and stored at −80°C.

### RNA Pull-Down

T7 RNA polymerase (Roche, Switzerland) was applied to reverse transcribe the full-length sequence of TCONS_00145741 or the negative control sequence, and purified with the RNeasy Mini Kit (QIAGEN, United States) referring to the reagent manufacturer’s standard procedures. Subsequently, the biotinylated TCONS_00145741 was incubated with the lysate of BV2 cells, and the eluted protein was purified. Ultimately, a Western blot was applied to measure the proteins in the eluate.

### RNA Immunoprecipitation

Magna RIP™ RNA-binding protein immunoprecipitation kit (Millipore) was applied for RIP assays. Specifically, the 1 × 10^7^ BV2 cell lysate was incubated with the negative control anti-IgG (ab190475, Abcam), anti-JNK (sc-7345, 1:500, Santa Cruz Biotechnology), anti-DUSP1 (sc-373841, 1:500, Santa Cruz Biotechnology), and anti-DUSP6 (ab76310, 1:1000, Abcam) conjugated magnetic beads. Subsequently, the RNA in the immunoprecipitation was extracted and the expression of TCONS_00145741 was analyzed using qRT-PCR.

### RNA FISH Followed by Immunofluorescence Analysis

RNA FISH followed by immunofluorescence analysis was carried out as the previously described methods (Ni et al., 2019). The synthetic RNA FISH probes were designed. Briefly, microglia were fixed with 4% formaldehyde for nearly 15min. The cells were then further incubated with FISH probes in a hybridization buffer. After hybridization, the plates were dehydrated and the nuclear DNA was labeled with DAPI. Fluorescence microscopy was applied to observe the slices with immunofluorescence.

### Construction of a Model of ICH Interfered With TCONS_00145741

Forty male C57BL/6 mice were applied to construct the model of ICH interfered with TCONS_00145741 and were randomly divided into these groups: vehicle (24 h), Sh-741 (24 h), vehicle (72 h), and Sh-741 (72 h) group, and each group was assigned 10 mice. For the mice in the vehicle (24 h) and vehicle (72 h) group, 3 μl AAV-NC (1 × 10^9^ TU/ml) was injected into the cerebral ventricle 2 day before ICH modeling, and they were sacrificed after ICH 24 h and ICH72 h. For the mice in the Sh-741 (24 h) and Sh-741 (72 h) groups, 3 μl AAV-Sh-741 (1 × 10^9^ TU/ml) was injected into the cerebral ventricle 2 day before the establishment of the ICH model, and they were sacrificed after ICH 24 h and ICH 72 h. All animal assays were approved by the Animal Care and Use Committee of the Second Affiliated Hospital of Nanchang University, Institute of Neuroscience, Nanchang University.

### Neurologic Deficit Score

At 24 and 72 h after ICH, we evaluated the neurological injury of mice through a 24-point system as the previously described methods (Cheng et al., 2016). The higher the score, the more severe the nerve injury. Besides, all tests were conducted blindly.

### Statistical Analysis

All the data were presented as mean ± standard deviation, and all the statistical analyses were conducted using SPSS 20.0 (IBM, NY) or GraphPad Prism 6.05 (GraphPad, CA). The statistical significance was assessed by Student’s *t*-test (the differences between the two groups) and one-way ANOVA (the differences among more than two groups). *p* < 0.05 was considered significant.

## Results

### Construction of Mouse Model of Acute Cerebral Hemorrhage and the Activation of Microglia in the Injured Area of Mice With Acute Intracerebral Hemorrhage

**FIGURE 1:**
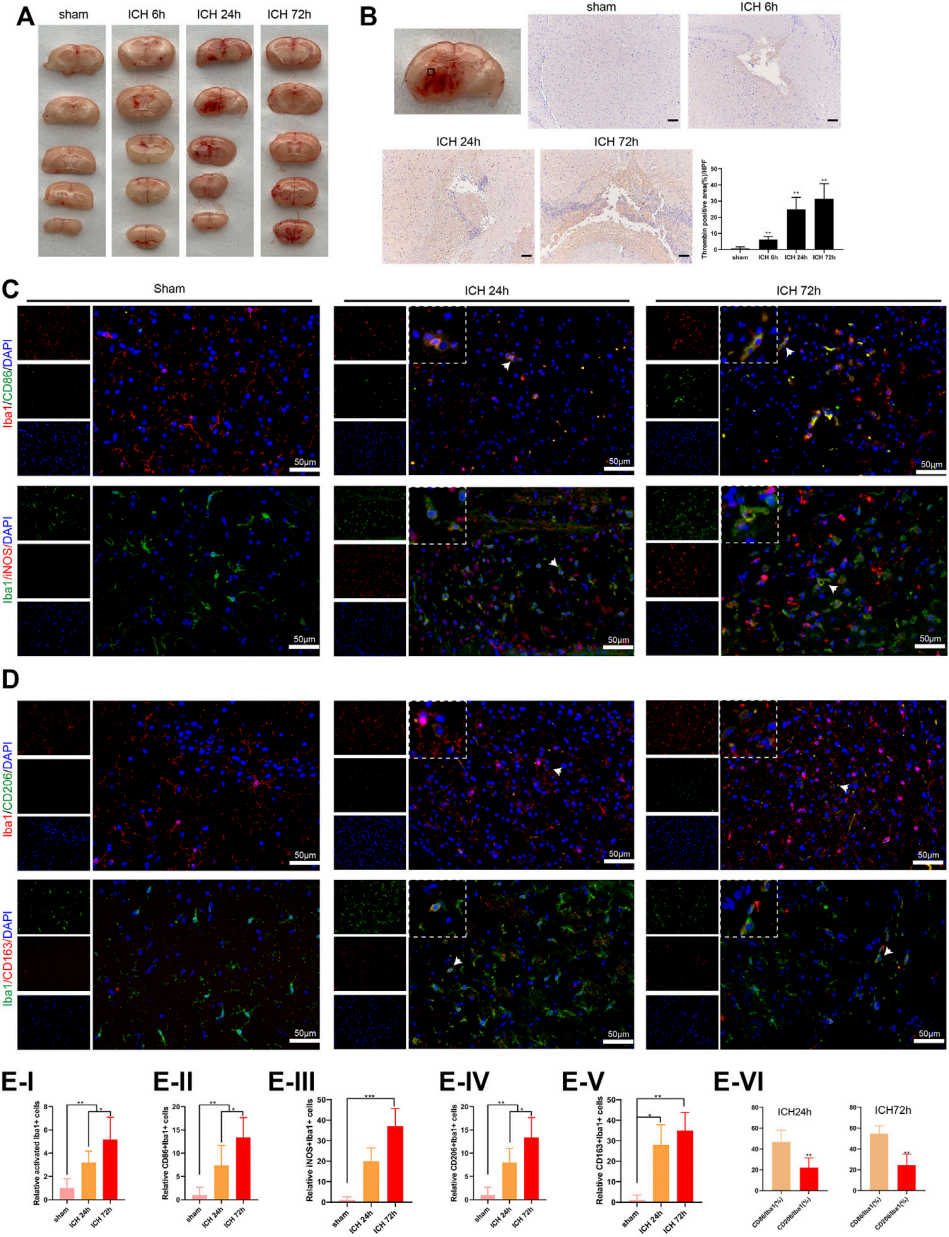
Activation of microglia in the injured area of mice with acute intracerebral hemorrhage. **(A)** Coronal section of hematoma to observe the bleeding in the brain of mice, n = 6 (Scale bar = 100 μm). **(B)** Detection of thrombin by immunohistochemistry assay, n = 6. **(C–E)** Immunofluorescence analysis was conducted to observe the activation of mouse microglia, including the relative activated Iba1+ cells, relative CD86+/Iba1+ cells, iNOS+/Iba1+ cells, CD206+/Iba1+ cells and CD163+/Iba1+ cells, n = 6 (Scale bar = 50 μm). *p < 0.05 vs. sham or ICH 24 h **p < 0.01 vs. sham or CD86/Iba1 (%). ***p < 0.001 vs. sham. ICH, intracerebral hemorrhage.

Here, we assessed the activation of microglia and the number of M1 and M2 microglia to verify the effect of M1 differentiation of microglia on ICH. As presented in Figure 1A, there was a bleeding site on the side of the brain of the ICH group of mice, and the bleeding area reached the maximum after 24 h; and the bleeding area was reduced after 72 h. Furthermore, the thrombin was gradually elevated as the increased bleeding time, and thrombin was accumulated near the bleeding point in the ICH group (Figure 1B). Iba1 is a microglia/macrophage-specific protein (Ohsawa et al., 2004), and CD86, iNOS are known as M1 marker; CD206, CD163 are known as M2 marker (Wang et al., 2020). Immunofluorescence assay showed that the number of M1 and M2 microglia was elevated with the increased bleeding time; compared with M1 microglia, the number of M2 microglia was decreased (Figures 1C–E). The above experimental data revealed that thrombin was elevated in ICH mice around ICH, and both M1 and M2 microglia were elevated, while the number of M2 microglia was less than that of M1 microglia.

### LncRNA Expression Profiles in Thrombin-Induced Primary Microglia and Verification of Differentially Expressed lncRNAs

**FIGURE 2:**
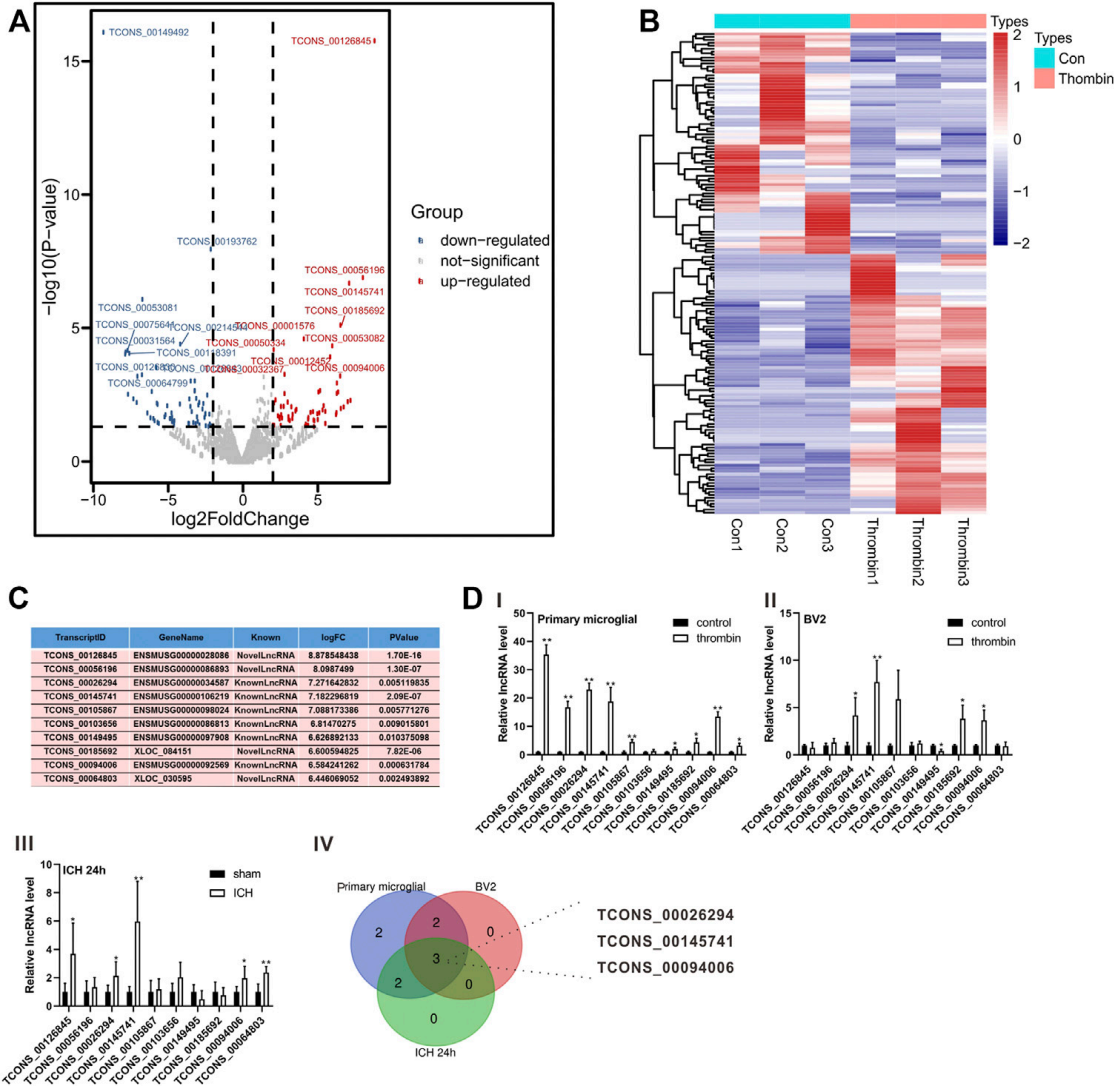
Expression profiles of lncRNAs induced by thrombin in primary microglia. **(A)** The Scatter plot compiled differentially expressed lncRNAs between the thrombin group and the control group. **(B)** Hierarchical clustering analysis of the differentially expressed lncRNAs between the thrombin group and the control group. **(C)** The top 10 up-regulated lncRNAs with the highest fold change between the thrombin group and the control group. **(D)** Detection of the above 10 differential lncRNAs in thrombin-treated primary microglia, thrombin-treated BV2 cells, and the ipsilateral coronal area of the ICH 24 h group by quantitative real-time PCR (qRT-PCR). *p < 0.05, **p < 0.01 vs. control or sham.

Thrombin facilitates secondary injury after ICH (Krenzlin et al., 2020) and thrombin regulates the polarization of microglia (Tao et al., 2016). Thus, we used the *in vitro* thrombin toxicity model applied to microglia to study the lncRNA and mRNA expression profiles of microglia activated by thrombin. As presented in Figure 2A, a total of 1,427 lncRNAs were differentially expressed between the thrombin group and the control group, including 762 elevated lncRNAs and 665 decreased lncRNAs. Hierarchical clustering analysis was conducted for the differentially expressed lncRNAs (Figure 2B). On this basis, we selected the top 10 elevated lncRNAs with the highest fold change between the thrombin group and the control group for subsequent detection (Figure 2C). Next, we performed qRT-PCR verification on the above 10 differential lncRNAs and tested them in the thrombin-treated primary microglia, thrombin-treated BV2, and the ipsilateral coronal area of the ICH 24 h group. The results illustrated that the change trends of TCONS_0026294, TCONS_00145741, and TCONS_00094006 were the same, and there were significant differences (Figure 2D). Thus, TCONS_0026294, TCONS_00145741, and TCONS_00094006 were selected for follow-up investigations.

### LncRNA TCONS_00145741 Regulates the Activation of M1 and M2 Microglia Under Thrombin Treatment

**FIGURE 3:**
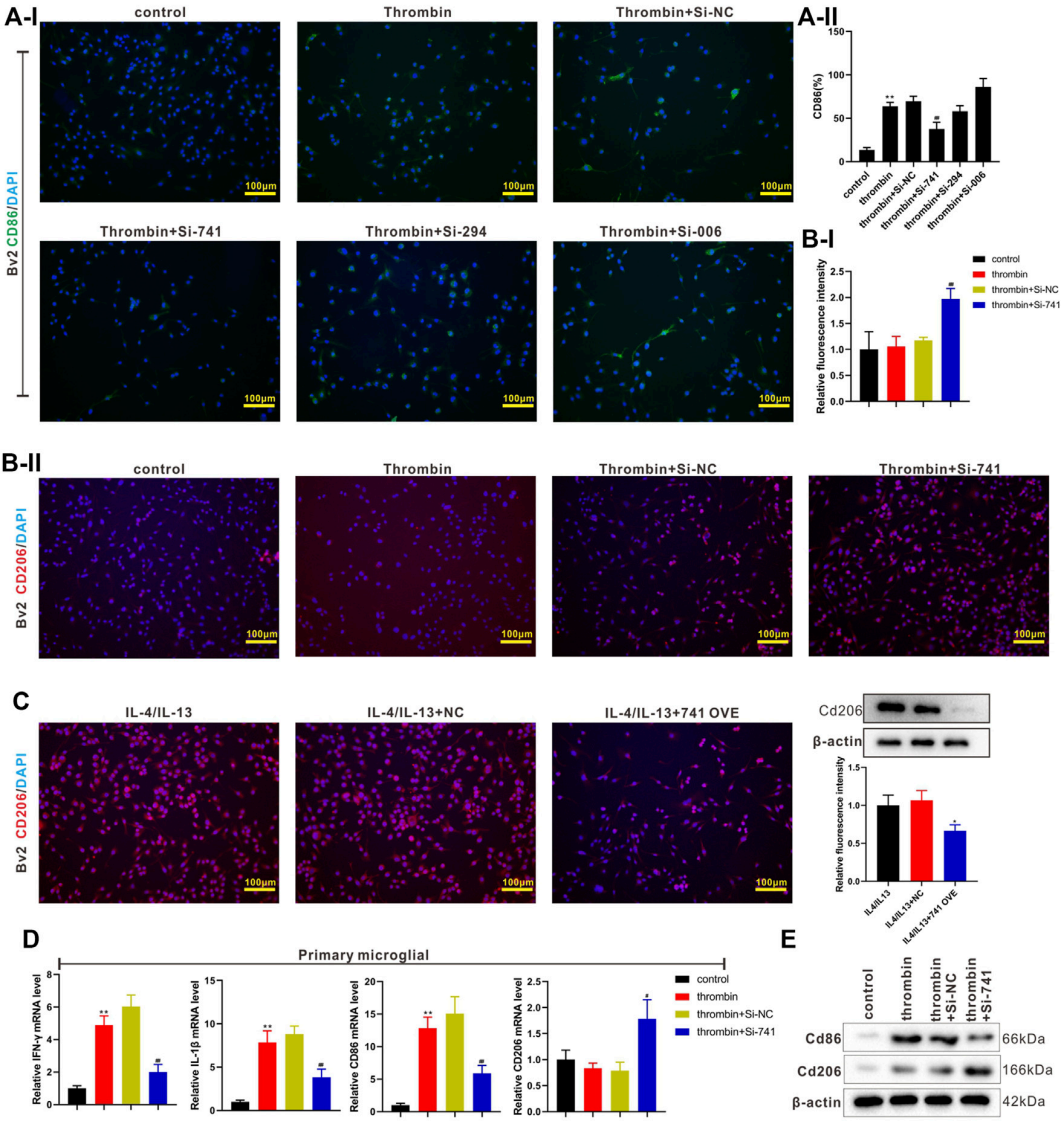
Effect of TCONS_00145741 on the activation of M1 and M2 microglia under thrombin treatment. **(A)** si-741, si-294, or si-006 was transfected into BV2 cells and then the cells were treated with 20 U/ml thrombin for 24 h. An immunofluorescence assay was carried out to assess the expression of CD86 in BV2 cells (Scale bar = 100 μm). **(B)** si-741was transfected into BV2 cells and then the cells were treated with 20 U/ml thrombin for 24 h. An immunofluorescence assay was conducted to analyze the expression of CD206 in BV2 cells (Scale bar = 100 μm). **(C)** 741 OVE (overexpression of 741) was transfected into BV2 cells and then the cells were treated with 10 ng/ml interleukin (IL)-4 and 10 ng/ml IL-13 for 24 h. Analysis of the CD206 in BV2 cells using immunofluorescence (Scale bar = 100 μm) and Western blot assays. **(D)** si-741was transfected into primary microglia and then treated the cells with 20 U/ml thrombin for 24 h. Detection of IFN-gamma, IL-1beta, CD86, and CD206 expressions in primary microglia by qRT-PCR. **(E)** Analysis of CD86 and CD206 protein levels by Western blot. *p < 0.05 vs. IL-4/IL-13 + NC. **p < 0.01 vs. control. ^#^ *p* < 0.05, ^##^ *p* < 0.01 vs. thrombin + si-NC. NC: negative control, IL-4/IL-13: the cells were treated with IL-4 and IL-13, 741: TCONS_00145741, 294: TCONS_0026294, 006: TCONS_00094006.

Subsequently, we transfected si-TCONS_00145741 (abbreviated as si-741), si-TCONS_0026294 (abbreviated as si-294), and si-TCONS_00094006 (abbreviated as si-006) into BV2 cells and then treated the cells with thrombin. The results revealed that the thrombin treatment facilitated the M1 differentiation of microglia, while this facilitation was reversed after the transfection of si-741. Thus, TCONS_00145741 was selected for subsequent research (Figure 3A). Besides, the ensemble of 741 is Ensmusg00000106219 and located on chromosome 5 and the full-length is 2630 bp (Supplementary Figure S1A). Furthermore, the thrombin treatment had no obvious changes on the CD206 expression, while CD206 was elevated after the transfection of si-741 (Figure 3B), implying that the transfection of si-741 facilitated the M2 differentiation of microglia under thrombin treatment.

IL-4 and IL-13 are commonly used to induce the M2 differentiation of microglia (Littlefield and Kohman, 2017). Next, 741 OVE (the overexpression of 741) was transfected into BV2 cells and then the cells were treated with IL-4 and IL-13. As displayed in Figure 3C, the 741 overexpression lessened the CD206 expression, hinting that the 741 overexpression restrained the M2 differentiation of microglia. Meanwhile, the analysis of CD86 and CD206 expressions using flow cytometry displayed the same trend (Supplementary Figure S2). Besides, si-741 was transfected into primary microglia and then the cells were treated with thrombin. As presented in Figures 3D,E, the transfection of si-741 reduced the expression of the M1 markers (IFN-gamma, IL-1beta, CD86), and elevated M2 marker CD206, implying that the interference with lncRNA TCONS_00145741 restrained the M1 differentiation of microglia and facilitated the M2 differentiation of microglia under thrombin treatment.

### TCONS_00145741 Regulates the Activation of the JNK Pathway in Microglia Under Thrombin Treatment

**FIGURE 4:**
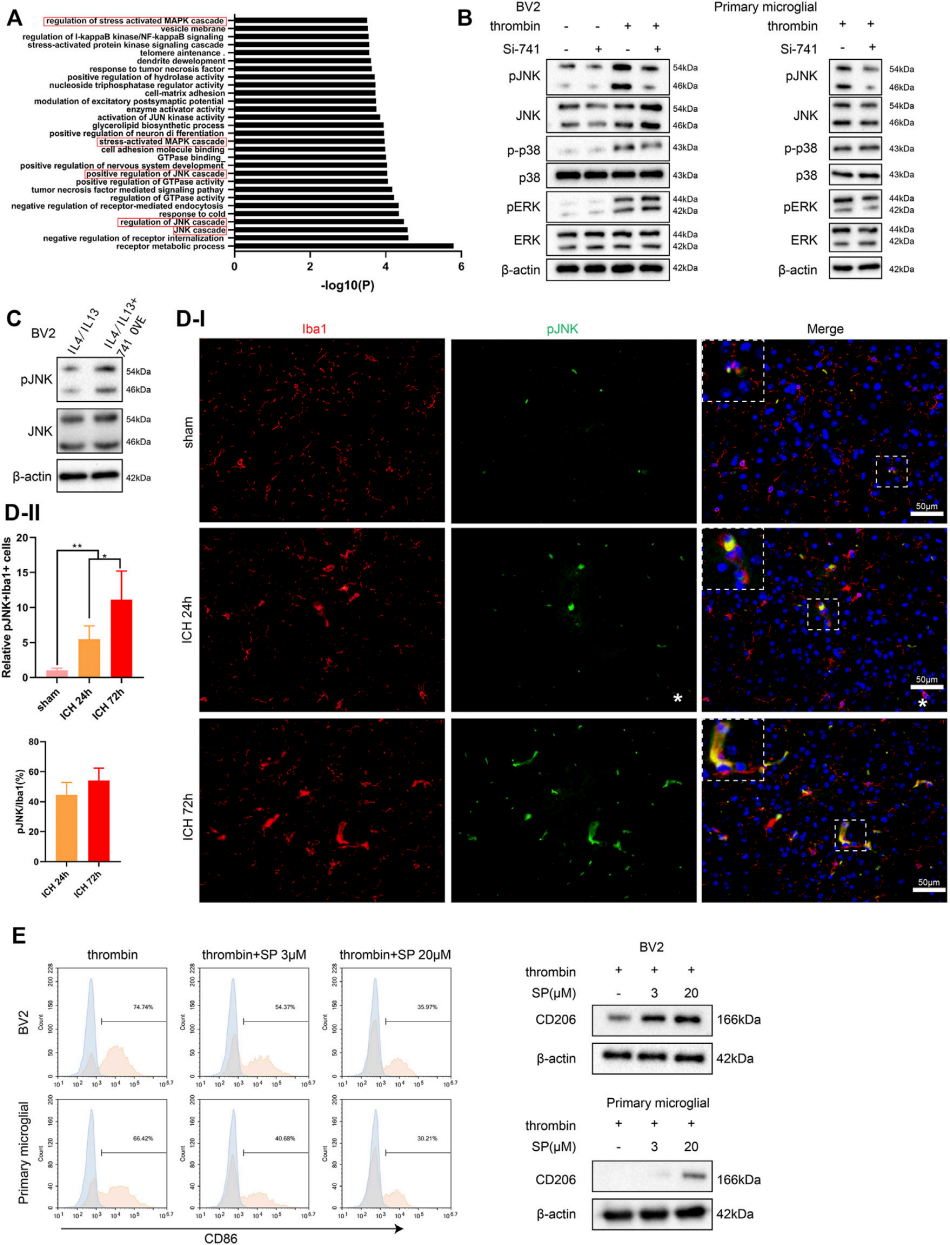
Influence of TCONS_00145741 on the activation of the JNK pathway in microglia under thrombin treatment. **(A)** Gene Ontology (GO) analysis of mRNA related to TCONS_00145741 in the sequencing results. **(B)** si-741 was transfected into BV2 cells and primary microglia and then treated the cells with 20 U/ml thrombin for 24 h. Detection of p-JNK, JNK, p-p38, p38, pERK, and ERK protein levels by Western blot. **(C)** 741 OVE was transfected into BV2 cells and then treated the cells with 10 ng/ml IL-4 and 10 ng/ml IL-13 for 24 h. Detection of p-JNK and JNK protein levels using Western blot. **(D)** Iba1/p-JNK double staining of the area around hematoma *in vivo* (Scale bar = 50 μm). **(E)** After treating BV2 cells and primary microglia with thrombin, the cells were treated with 3 μM or 20 μM JNK pathway inhibitor SP600125. Detection of CD86 by flow cytometry and analysis of CD206 protein level by Western blot. **p < 0.01 vs. sham. *p < 0.05 vs. ICH 24 h. Si-741: si-TCONS_00145741, 741 OVE: the overexpression of TCONS_00145741, SP: SP600125.

Furthermore, we conducted a Gene Ontology analysis on the mRNAs related to TCONS_00145741 in the sequencing results and found that the MAPK signaling pathway was the most relevant (marked by the red box) (Figure 4A). Thus, the MAPK signaling pathway was chosen as a follow-up key pathway. Next, we transfected si-741 into BV2 cells and primary microglia and then treated or untreated the cells with thrombin. As displayed in Figure 4B, the thrombin treatment activated the JNK signaling pathway in microglia, and silencing 741 reduced its activation. Under the condition of IL4/IL13 treatment of BV2 cells, the 741 overexpression enhanced the p-JNK expression (Figure 4C). Besides, Iba1/p-JNK double staining of the area around hematoma *in vivo* corroborated that p-JNK was elevated in microglia under ICH conditions (Figure 4D). After treating BV2 cells and primary microglia with thrombin, the cells were treated with different concentrations of JNK pathway inhibitor SP600125. As displayed in Figure 4E, when elevating the concentration of SP600125 to strengthen the JNK inhibitory ability, the ability of thrombin to facilitate CD86 was gradually weakened, and the expression of CD206 was gradually elevated, implying that the JNK pathway was interrelated to the activation of thrombin on microglia.

### TCONS_00145741 Stabilizes JNK Phosphorylation Levels Through Disruption of the Interaction Between DUSP6 and JNK

**FIGURE 5:**
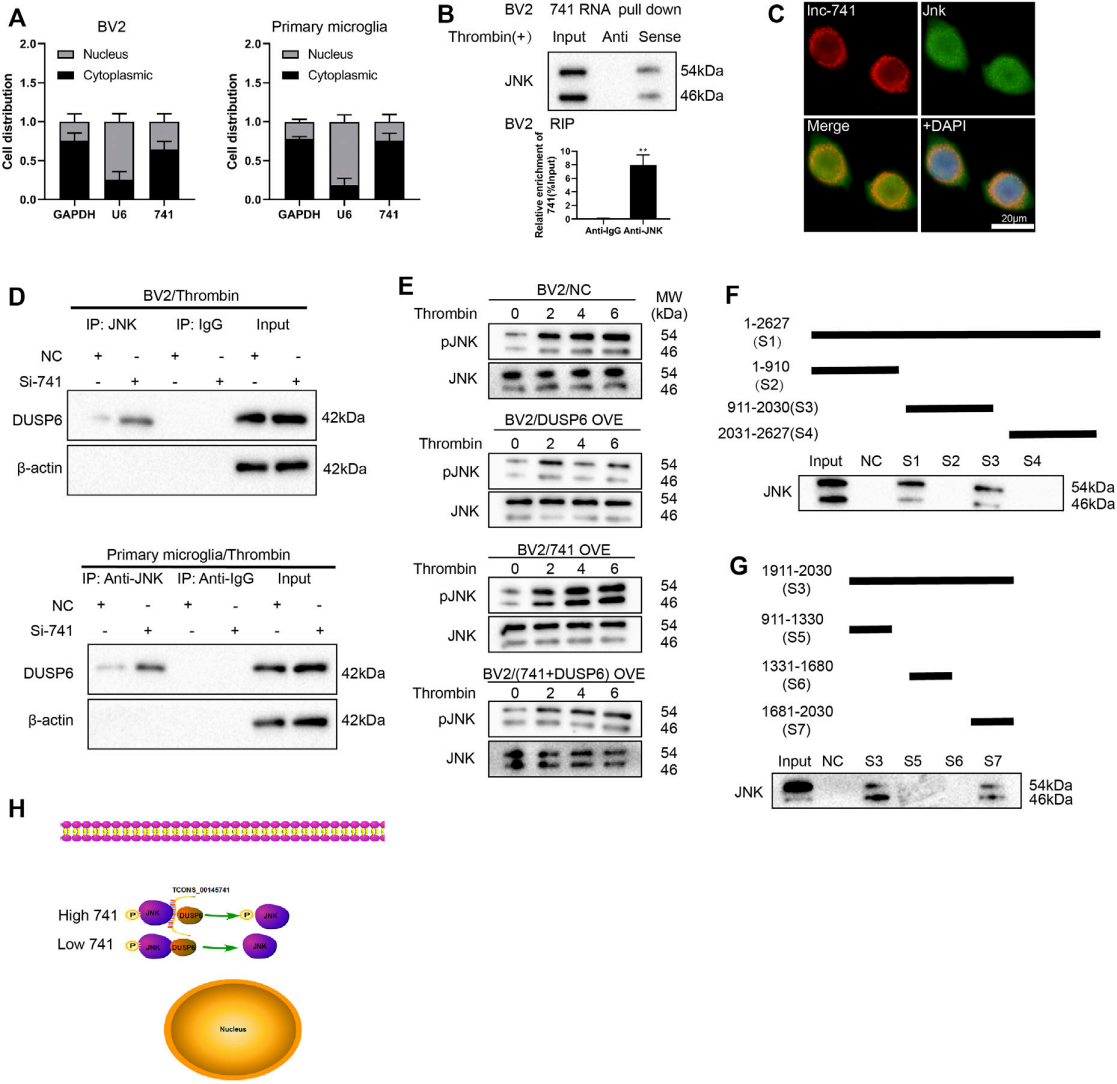
TCONS_00145741 regulates the level of JNK phosphorylation by disrupting the interaction between DUSP6 and JNK. **(A)** BV2 cells and primary microglia were treated with 20 U/ml thrombin for 24 h. Detection of TCONS_00145741 expression in the cytoplasm and nucleus of cells by qRT-PCR. **(B)** RNA pull-down and RNA immunoprecipitation assays were carried out to verify that TCONS_00145741 combined with JNK protein. **(C)** RNA FISH followed by immunofluorescence analysis was applied to analyze the co-localization of TCONS_00145741 and JNK in microglia (Scale bar = 20 μm). **(D)** TCONS_00145741 was silenced in BV2 cells and primary microglia and then the cells were treated with 20 U/ml thrombin for 24 h. Detection of the protein level of DUSP6 by Western blot. **(E)** TCONS_00145741 and/or DUSP6 were overexpressed in BV2 cells and then the cells were treated with thrombin for 0, 2, 4, and 6 h. Detection of the protein levels of pJNK and JNK. **(F–H)** A series of TCONS_00145741 truncated segments were designed to clarify the binding sites of TCONS_00145741 that interacted with JNK. **p < 0.01 vs. Anti-IgG.

First of all, we extracted RNA from the nucleus and cytoplasm of BV2 cells and primary microglia after thrombin treatment and the quantified results demonstrated that TCONS_00145741 was elevated in the cytoplasm, implying that TCONS_00145741 was mainly located in the cytoplasm (Figure 5A). Considering that lncRNAs bind to miRNAs as competitive endogenous RNAs (ceRNAs) and thus function as miRNA sponges in cells, we carried out the AgO2-RIP assay and corroborated that TCONS_00145741 did not combine with AgO2 (Supplementary Figure S1B), thus we speculated that TCONS_00145741 might regulate intracellular signaling in other ways. As we all know, lncRNAs function in various diseases through binding proteins. Thus, we next carried out an RNA pull-down assay and demonstrated that TCONS_00145741 was directly combined with JNK protein (Figure 5B), and this conclusion was further confirmed by RIP assay (Figure 5B). Meanwhile, RNA FISH followed by immunofluorescence analysis corroborated that TCONS_00145741 co-localized with JNK in the cytoplasm but not in the nucleus of microglia (Figure 5C). Based on this finding, we speculated whether the binding of TCONS_00145741 to JNK repressed the dephosphorylation level of JNK protein. Previous research confirms that the dephosphorylation level of JNK protein is mainly regulated by dual-specificity phosphatases (DUSPs) (Ha et al., 2019). We further clarified that the TCONS_00145741 affected the binding of JNK to which DUSPs and the results corroborated that silencing TCONS_00145741 mainly enhanced the binding of JNK to DUSP6 (Supplementary Figure S1C). Thus, DUSP6 was chosen for the follow-up study. Similarly, in the thrombin treatment condition, the TCONS_00145741 knockdown enhanced the binding of DUSP6 to JNK in BV2 cells and primary microglia (Figure 5D). Besides, the thrombin treatment showed an elevation of p-JNK in 6 h post-stimulation and the overexpression of DUSP6 slowed the elevation in p-JNK levels; the overexpression of TCONS_00145741 accelerated the elevation of p-JNK levels, and the elevated rate of p-JNK in cells overexpressing TCONS_00145741 and DUSP6 was slower than that in the DUSP6 overexpressing group (Figure 5E). All the above results expounded that TCONS_00145741 was interrelated to the dephosphorylation of JNK by DUSP6.

To investigate which sequence of TCONS_00145741 bound to JNK, a series of TCONS_00145741 truncated segments were designed to clarify the binding sites of TCONS_00145741 that interacted with JNK. Through the fractioning experiments, we authenticated that S7 (1681-2030) was the main binding position with JNK on TCONS_00145741 (Figures 5F–H).

Furthermore, we assessed the sequence alignment of S7 in the NONCODE database and corroborated the human lncRNA NONHSAT162889.1 with high homology (Supplementary Figure S3A). After the human microglia HMC3 were treated with thrombin, NONHSAT162889.1 expression was elevated, and the elevation rate was more than 80 (Supplementary Figure S3B). Meanwhile, NONHSAT162889.1 bound to JNK (Supplementary Figure S3C). Combined with the function of JNK, this long-chain molecule might be a potential target. In general, the above analysis corroborated that the study of the pathological mechanism in mice might open up a novel direction for pathogenesis and drug use in humans.

Meanwhile, we also confirmed that TCONS_00145741 bound to DUSP6 (Supplementary Figures S1D–E), implying that TCONS_00145741 not only repressed the dephosphorylation of JNK by DUSP6 through binding to JNK, but also restrained dephosphorylation of JNK by DUSP6 through binding to DUSP6. Thus, TCONS_00145741 might act as a scaffold to influence the interaction between DUSP6 and JNK.

### TCONS_00145741 Regulates the Degree of a Cerebral Hemorrhage in Mice

**FIGURE 6:**
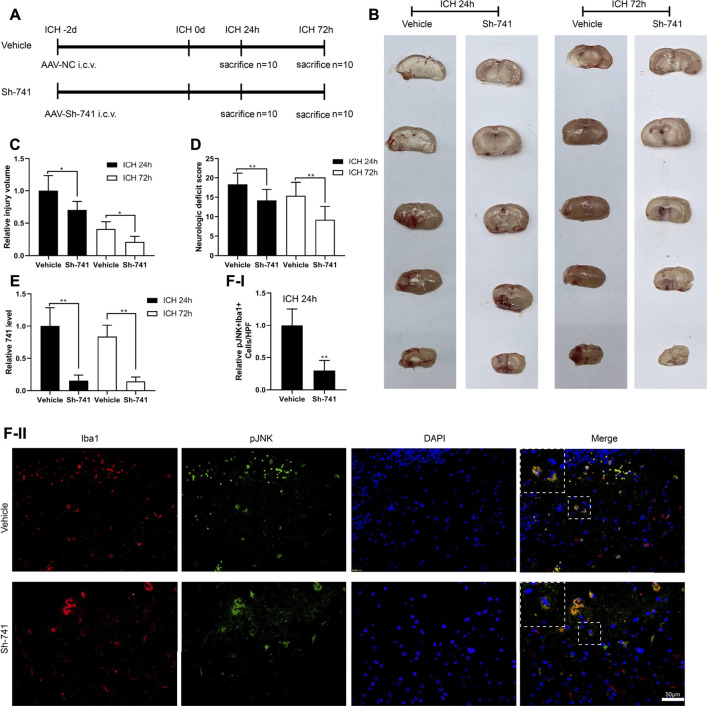
Effect of TCONS_00145741 on the degree of a cerebral hemorrhage in mice. **(A)** The experimental scheme for the construction of the mouse ICH model, n = 10. **(B and C)** Analysis of the injury volume in mice, n = 10. **(D)** Analysis of the neurologic deficit score, n = 10. **(E)** Detection of TCONS_00145741 expression by qRT-PCR, n = 10. **(F)** Detection of the pJNK using immunofluorescence, n = 10, (Scale bar = 50 μm). *p < 0.05, **p < 0.01 vs. vehicle. Sh-741: shRNA TCONS_00145741.

Moreover, we verified the effect of TCONS_00145741 in a mouse model of ICH. The experimental scheme for the construction of the mouse ICH model was displayed in Figure 6A. First, we assessed the injury volume in mice and corroborated that silencing TCONS_00145741 reduced the injury volume in mice (Figures 6B,C). Besides, behavioral testing expounded that silencing TCONS_00145741 reduced the neurologic deficit scores at 24 and 72 h after ICH (Figure 6D). qRT-PCR was carried out in the mouse injury area and nearby tissues and corroborated that TCONS_00145741 was decreased, implying that the TCONS_00145741 was successfully silenced (Figure 6E). Furthermore, under the premise of ICH 24 h, silencing TCONS_00145741 reduced the expression of pJNK in microglia (Figure 6F).

### TCONS_00145741 is Interrelated to the Activation of Microglia in ICH

**FIGURE 7:**
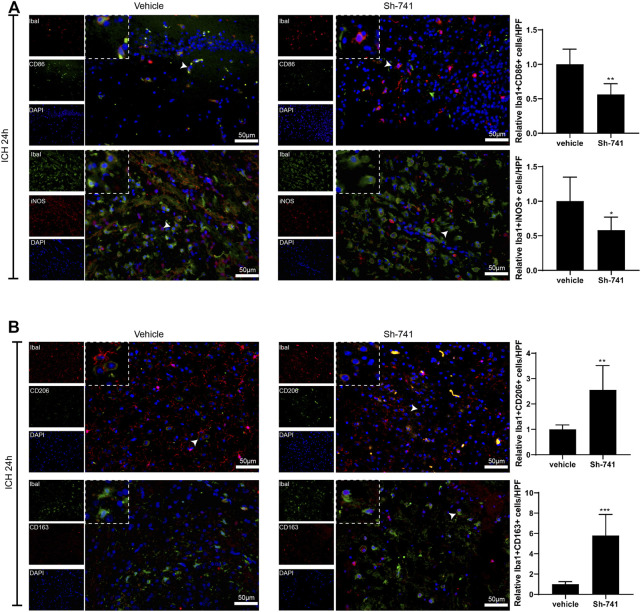
TCONS_00145741 affects the activation of microglia in ICH. **(A and –B)** 24 h after the establishment of the mouse ICH model, the activation of mouse microglia was assessed using an immunofluorescence assay (Scale bar = 50 μm). *p < 0.05, **p < 0.01, ***p < 0.001 vs. vehicle.

Subsequently, we delved into the regulation of 741 on the activation of microglia in ICH. As exhibited in Figure 7A-Figure 7B, 24 h after the establishment of the mouse ICH model, silencing 741 suppressed the M1 differentiation of microglia in mice subjected to ICH and facilitated the M2 differentiation of microglia. Meanwhile, 72 h after the construction of the mouse ICH model, the 741 knockdown restrained the M1 differentiation of microglia and promoted the M2 differentiation of microglia (Supplementary Figures S4A,B).

## Discussion

Increasing lncRNAs have been confirmed as biomarkers for neurological diseases including ICH (Wen et al., 2018; Zhou et al., 2019a; Villa et al., 2019), and the activation of microglia participates in the recovery process of brain injury after ICH (Lan et al., 2017a; Wang et al., 2018). In the current study, we applied RNA-sequencing to assess the changes in the expression profile of lncRNAs in microglia treated with thrombin, and selected the abnormally high expression and inflammation-related TCONS_00145741 as our research target and found that the interference with TCONS_00145741 restrained the activation of M1 microglia under thrombin treatment. Our further mechanism studies corroborated that the interference with TCONS_00145741 restrained JNK phosphorylation levels by enhancing the interaction between DUSP6 and JNK, finally showing a protective function for ICH mice. The mechanism of TCONS_00145741 mediating the pathological process of ICH is displayed in Supplementary Figure S5.

Previous studies illustrated that microglia function in ICH-induced nerve injury (Wan et al., 2016; Chen et al., 2019), and the imbalance of M1 and M2 differentiation of microglia after ICH aggravates the process of ICH (Tschoe et al., 2020). Similarly, our experimental data corroborated that in the ICH mouse model, the number of microglia was elevated with the prolongation of ICH time and the number of M1 microglia was higher than that of the M2. Recently, the abnormal expressions of lncRNAs in ICH have attracted widespread attention, and various studies attempt to provide novel potential biomarkers for ICH (Hanjin et al., 2018; Kim et al., 2019). Here, we analyzed the changes in the expression profile of lncRNAs in thrombin-treated microglia through RNA-sequencing and authenticated that TCONS_00145741 was elevated, and we further confirmed that the interference with TCONS_00145741 restrained the M1 differentiation of microglia treated with thrombin and facilitated the M2 differentiation of microglia.

MAPK signaling pathway mainly includes JNK, ERK, p38 MAPK, and other signaling pathways (Qiu et al., 2020), in which JNK and P38 are interrelated to inflammatory response and apoptosis, while ERK is interrelated to cell growth and differentiation (Yang et al., 2016). Besides, the phosphorylation of the MAPK signaling pathway was rapidly elevated in the acute ICH rat model (Ni et al., 2015) and primary rat microglia treated with thrombin (Akaishi et al., 2020). In the current study, we corroborated that TCOS_00145741 was interrelated to the MAPK signaling pathway and the NF-κB signaling pathway (Figure 4A). MAPK signaling pathway induces the occurrence of the NF-κB signaling pathway (Gaire et al., 2018; Subedi et al., 2019). Both NF-κB (Zeng et al., 2017) and MAPK signaling pathways (Wen et al., 2018) play momentous regulatory functions in microglia activation and ICH. Moreover, our data expounded that the interference with TCOS_00145741 reduced the activation of the JNK MAPK pathway in microglia treated with thrombin. The JNK MAPK signaling pathway is pro-inflammatory (Zhou et al., 2019b) and activates the downstream NF-κB signaling pathway (Chen et al., 2018). In the process of microglia differentiation, the JNK MAPK signaling pathway facilitates the M1 differentiation of microglia (Zhang et al., 2018a; Gaire et al., 2019), and the restraint of JNK activity in macrophages facilitates the M2 differentiation of macrophages (Zhou et al., 2019c; Zhang et al., 2019). Interestingly, our results also demonstrated that the restraint of the JNK pathway weakened the M1 differentiation ability of thrombin against microglia and enhanced the M2 differentiation ability, hinting that the JNK pathway was interrelated to regulate the activation process of thrombin against microglia.

Phosphorylated MAPK molecules activate the subsequent signaling pathways, so the phosphorylation level of MAPK molecules is a momentous basis for judging the activity of MAPK signaling pathways. Studies indicate that various lncRNAs mediate the phosphorylation of proteins, such as restraining the phosphorylation of proteins (Zhang et al., 2018b; Jin et al., 2019) or facilitating the phosphorylation of proteins (Xu et al., 2020). Dual specificity phosphatases (DUSPs) bind to the MAPK molecule p38, ERK1/2, and JNK to dephosphorylate the MAPK molecules (Ha et al., 2019), thereby restraining the activity of JNK. In this study, we also authenticated that TCOS_00145741 stabilized JNK phosphorylation levels through disruption of the interaction between DUSP6 and JNK.

In summary, our study demonstrated that the interference with lncRNA TCONS_00145741 ameliorated ICH by restraining thrombin-induced M1 differentiation of microglia, supporting that lncRNA TCONS_00145741 might function as a novel target for ICH treatment. Our study might provide novel biomarkers for relieving ICH. Importantly, the study of this pathological mechanism in mice might open up a new direction for pathogenesis and drug use in humans.

## Data Availability

The datasets presented in this study can be found in online repositories. The names of the repository/repositories and accession number(s) can be found below: The NCBI accession numbers are SRR14883722, SRR14883725, SRR14883727, SRR14883726, SRR14883724 and SRR14883723. The BioSample accessions are: SAMN19819898, SAMN19819899, SAMN19819900, SAMN19819901, SAMN19819902 and SAMN19819903. The Bioproject number is PRJNA740100.

## Supplementary Material

Supplementary Figure S1
**(A)** Information of 741 sequences. **(B)** The agO2-RIP assay was conducted to assess the binding of TCONS_00145741 to AgO2. **(C)** Immunoprecipitation was applied to clarify the TCONS_00145741 affected the binding of JNK to which DUSPs. (D-E) Verification of the binding of 741 to DUSP6. ***P* < 0.01 vs. IgG.

Supplementary Figure S2 Analysis of the impact of TCONS_00145741 in the activation of M1 and M2 microglia under thrombin treatment. **(A)** si-741was transfected into BV2 cells and then the cells were treated with 20 U/ml thrombin for 24 h; 741 OVE was transfected into BV2 cells and then the cells were treated with 10 ng/ml IL-4 and 10 ng/ml IL-13 for 24 h. Detection of the CD206 in BV2 cells using flow cytometry. **(B)** si-741, si-294, or si-006 was transfected into BV2 cells and then the cells were treated with 20 U/ml thrombin for 24 h. The expression of CD86 was assessed by flow cytometry. *****P* < 0.0001 vs. control, thrombin + si-NC, IL-4/IL-13 + NC.

Supplementary Figure S3
**(A)** NONCODE database was performed to analyze the sequence alignment of S7 and found the human lncRNA NONHSAT162889.1 with high homology. **(B)** After the human microglia HMC3 were treated with thrombin, NONHSAT162889.1 expression was measured by qRT-PCR. **(C)** Verification of the binding of NONHSAT162889.1 to JNK. ***P* < 0.01 vs. control.

Supplementary Figure S4 Impact of TCONS_00145741 on the activation of microglia in ICH. **(A and B)** 72 h after the construction of the mouse ICH model, the activation of mouse microglia was assessed by immunofluorescence (Scale bar = 50 μm). **P* < 0.05, ***P* < 0.01 vs. vehicle.

Supplementary Figure S5 The mechanism of TCONS_00145741 mediates the pathological process of ICH. Under ICH pathological conditions, the expression of TCONS_00145741 was elevated after thrombin treatment of microglia, and the highly expressed TCONS_00145741 elevated JNK phosphorylation levels by restraining the binding of DUSP6 and JNK, which in turn led to the secretion of pro-inflammatory factors and the differentiation of microglia from M0 to M1, and further imbalanced M1/M2 to eventually aggravate ICH.
