# Supplementary figures and images for: LncRNA TCONS_00145741 Knockdown Prevents Thrombin-Induced M1 Differentiation of Microglia in Intracerebral Hemorrhage by Enhancing the Interaction Between DUSP6 and JNK
Source: Front Cell Dev Biol. 2022 Jan 19;9:684842. doi: 10.3389/fcell.2021.684842 (PMC8809462; doi:10.3389/fcell.2021.684842)

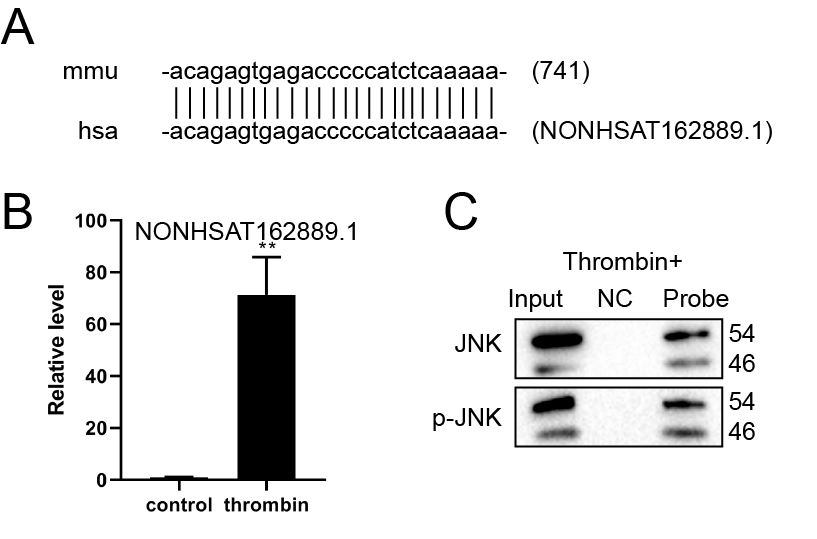

Supplement: Supplementary file 1 [file Image3.JPEG]

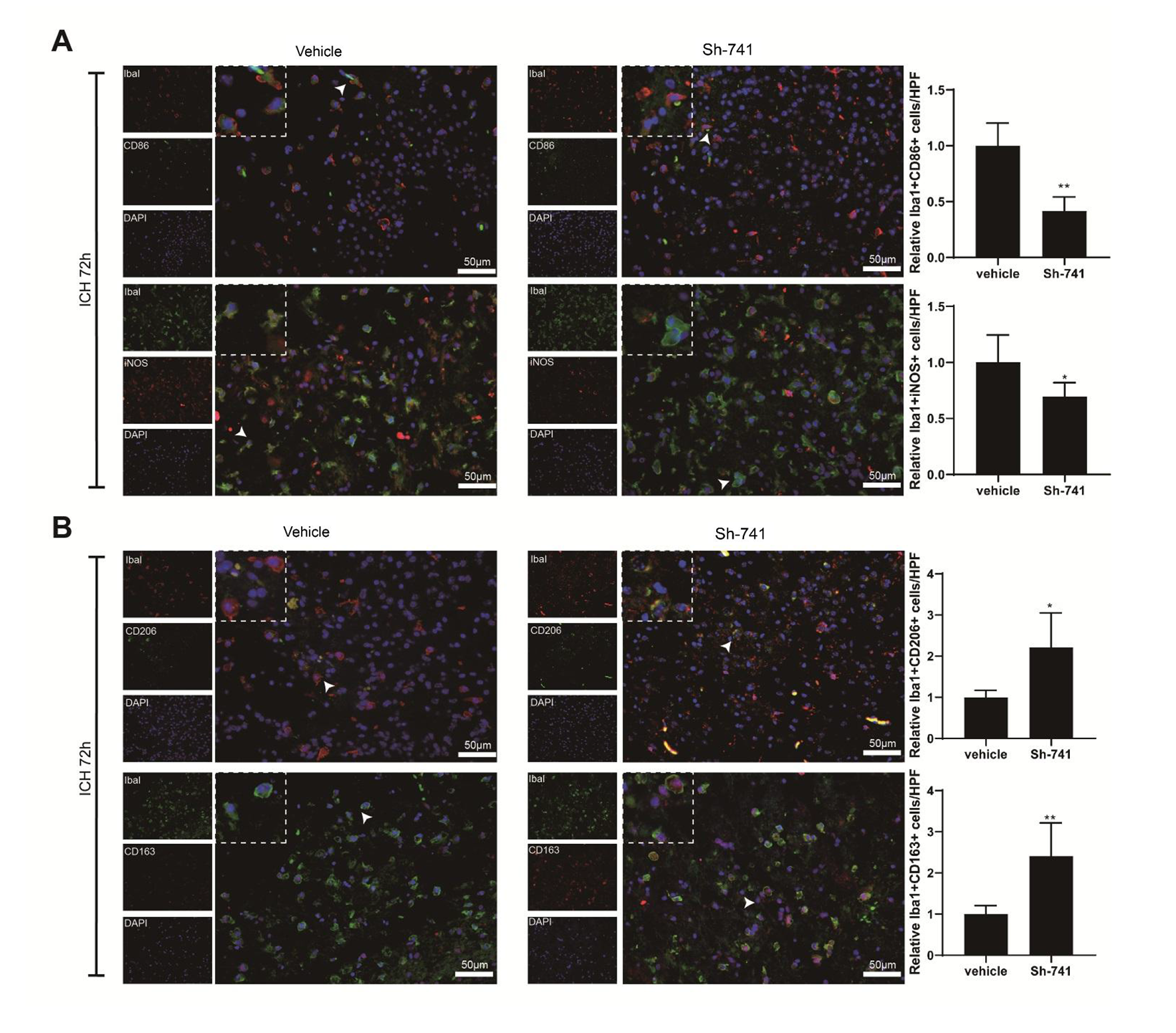

Supplement: Supplementary file 2 [file Image4.TIF]

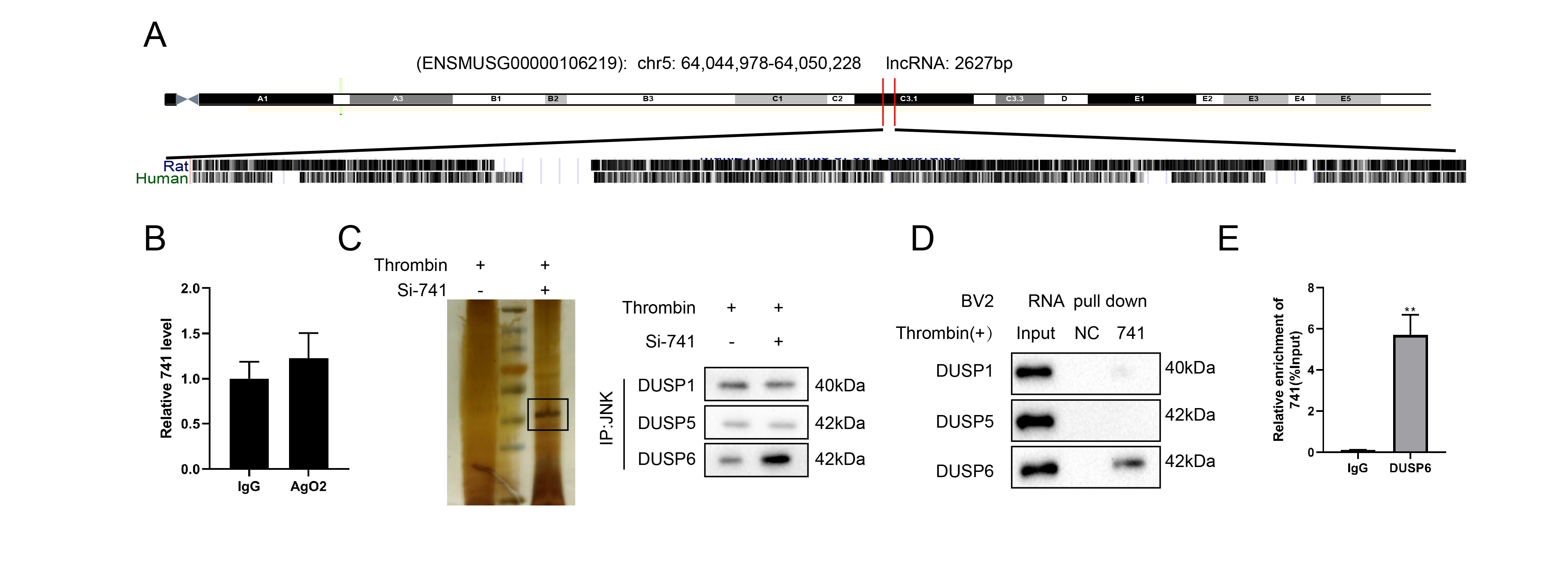

Supplement: Supplementary file 3 [file Image1.JPEG]

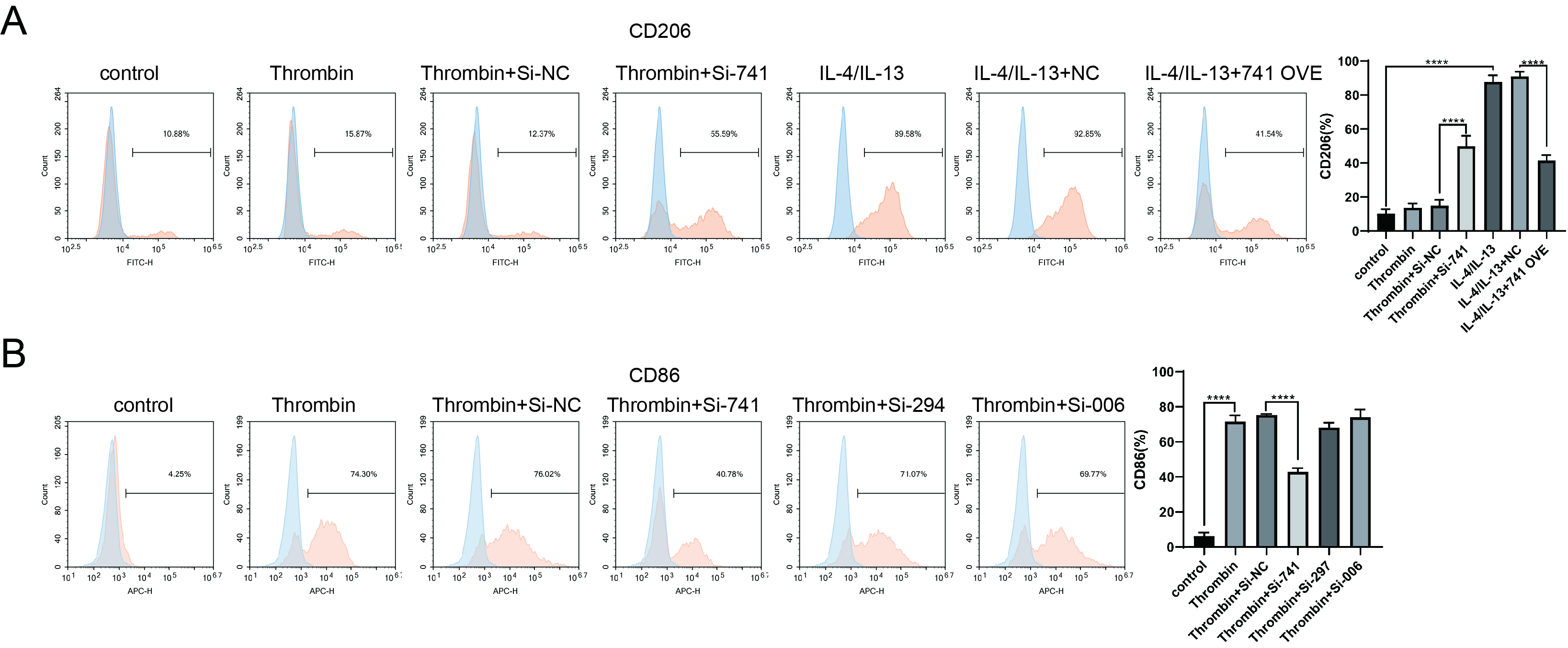

Supplement: Supplementary file 4 [file Image2.JPEG]

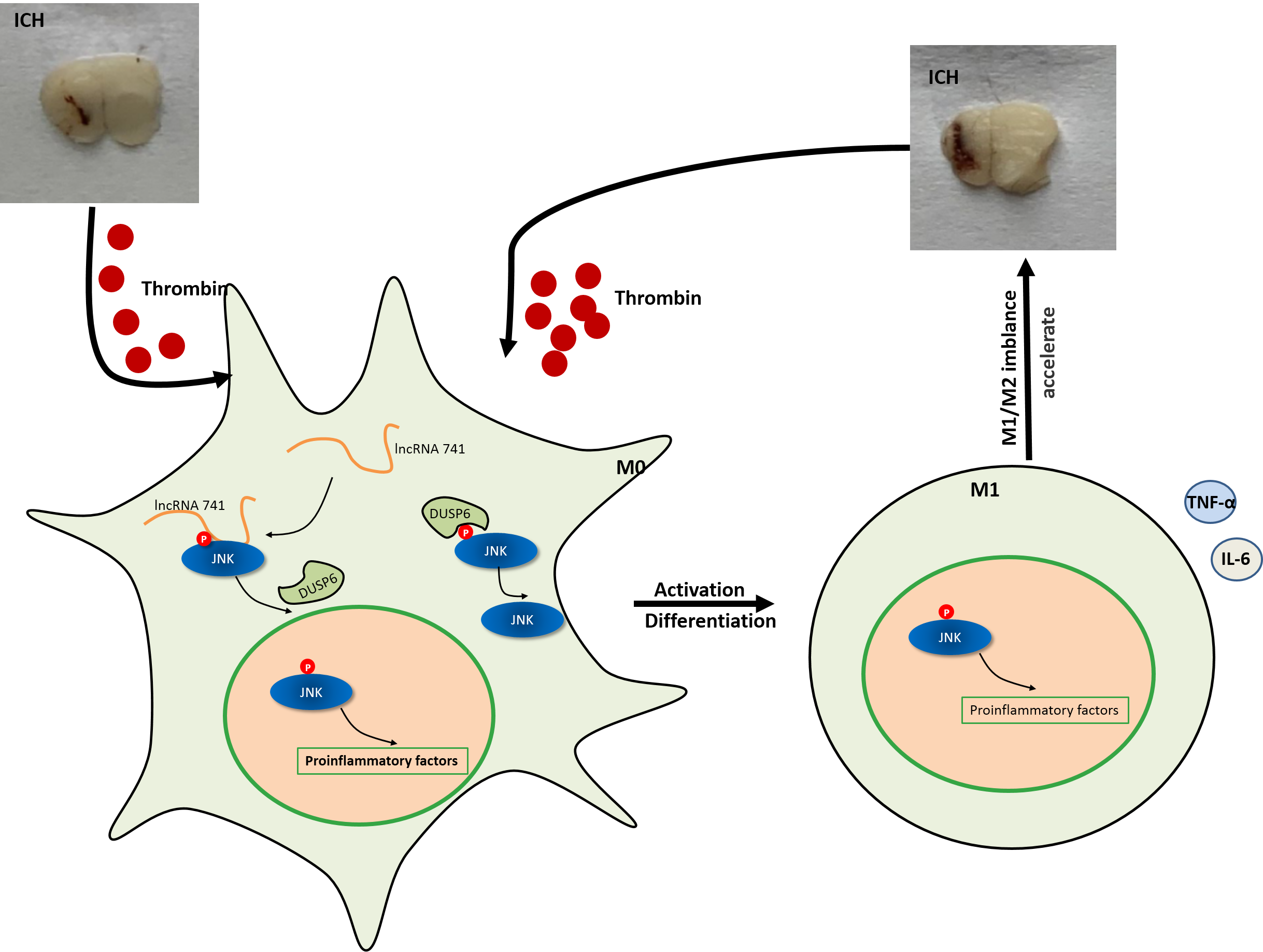

Supplement: Supplementary file 5 [file Image5.JPEG]
